# A Nomogram Combined Radiomics and Clinical Features as Imaging Biomarkers for Prediction of Visceral Pleural Invasion in Lung Adenocarcinoma

**DOI:** 10.3389/fonc.2022.876264

**Published:** 2022-05-25

**Authors:** Xinyi Zha, Yuanqing Liu, Xiaoxia Ping, Jiayi Bao, Qian Wu, Su Hu, Chunhong Hu

**Affiliations:** ^1^ Department of Radiology, The First Affiliated Hospital of Soochow University, Suzhou, China; ^2^ Institute of Medical Imaging, Soochow University, Suzhou, China

**Keywords:** CT, lung adenocarcinomas, radiomics, Nomogram, prediction, visceral pleural invasion

## Abstract

**Objectives:**

To develop and validate a nomogram model based on radiomics features for preoperative prediction of visceral pleural invasion (VPI) in patients with lung adenocarcinoma.

**Methods:**

A total of 659 patients with surgically pathologically confirmed lung adenocarcinoma underwent CT examination. All cases were divided into a training cohort (n = 466) and a validation cohort (n = 193). CT features were analyzed by two chest radiologists. CT radiomics features were extracted from CT images. LASSO regression analysis was applied to determine the most useful radiomics features and construct radiomics score (radscore). A nomogram model was developed by combining the optimal clinical and CT features and the radscore. The model performance was evaluated using ROC analysis, calibration curve and decision curve analysis (DCA).

**Results:**

A total of 1316 radiomics features were extracted. A radiomics signature model with a selection of the six optimal features was developed to identify patients with or without VPI. There was a significant difference in the radscore between the two groups of patients. Five clinical features were retained and contributed as clinical feature models. The nomogram combining clinical features and radiomics features showed improved accuracy, specificity, positive predictive value, and AUC for predicting VPI, compared to the radiomics model alone (specificity: training cohort: 0.89, validation cohort: 0.88, accuracy: training cohort: 0.84, validation cohort: 0.83, AUC: training cohort: 0.89, validation cohort: 0.89). The calibration curve and decision curve analyses suggested that the nomogram with clinical features is beyond the traditional clinical and radiomics features.

**Conclusion:**

A nomogram model combining radiomics and clinical features is effective in non-invasively prediction of VPI in patients with lung adenocarcinoma.

## Introduction

Lung cancer is currently the second most common cancer in the world and remains the leading cause of death among malignant tumors (1). Over 83% of lung cancers are non-small cell lung cancer (NSCLC) (2). Visceral pleural invasion (VPI), defined as tumor extension beyond the elastic layer of viscera pleura, is one of the most important adverse prognostic factors in non-small cell lung cancers with tumor sizes ≤ 3 cm (3, 4). In the eighth edition of TNM classification for NSCLC, VPI increases the T staging of lung cancer with diameters ≤ 3 cm: the presence of VPI leads to upstaging T1 tumor to T2 and stage IA tumor to IB (5, 6).

Several studies have evaluated the morphological characteristics of VPI in NSCLC based on CT images (7–9). However, there is no definite morphological feature that can reliably predict VPI, especially when the tumor is far from the pleura without pleura indentation or pleural attachment (7–9). Radiomics extracts a large amount of quantitative information from medical images (10, 11). Radiomics have been utilized for clinical-decision support systems in lung cancer, including diagnose and prognostic prediction (12–14). However, few studies have been reported to assess for the presence of VPI in patients with NSCLC using radiomics methods (15).

Therefore, the purpose of this study was to construct a nomogram model based on radiomics features, and determine whether VPI of lung adenocarcinoma can be predicted using the model.

## Materials and Methods

### Patients

This retrospective study was approved by the institutional review board of the First Affiliated Hospital of Soochow University (Suzhou, China), and the requirement for patient informed consent was waived. Patients with peripheral lung adenocarcinoma who underwent chest CT scans with thin-section (1–1.25 mm) images from January 2016 to December 2020 were reviewed. Inclusion criteria were as follows: (a) all the patients were confirmed as lung adenocarcinoma by pathological examination, and whether pleural invasion or not was evaluated pathologically; (b) the peripheral lesion was determined as N0M0 stage with the largest diameter smaller than 3.0 cm; (c) thin-section CT scan was performed within 30 days before surgery; (d) available results for clinical data, including age, sex, smoking history; (e) at least one of the following features were presented on CT images: pleural depression, pleural attachment or pleural closeness. 750 patients were excluded because of the following reasons: (a) histological diagnosis of SCLC (n=89); (b) tumor size > 3 cm (n=185); (c) whether pleural invasion or not cannot be assessed pathologically (n=136); (d) the lesion is far from the pleura without any of the above three features (n =301). (e) poor imaging quality due to respiratory artifact during examination (n=39). Finally, A total of 659 patients met all the inclusion criteria and included in this study.

### CT Scans

Patients underwent preoperative unenhanced CT scanning using various multidetector row scanners: Brilliance 16 or Brilliance iCT (Philips Healthcare, Best, the Netherlands), Somatom Sensation 64 or Somatom Definition (Siemens Healthineers, Erlangen, Germany), GE revolution or Discovery CT 750 HD (GE Healthcare, Chicago, USA), Aquilion One (Toshiba Medical Systems, Tokyo, Japan). The imaging parameters for thin-section CT were as follows: tube voltage 100-120 kV, automatic tube current modulation, matrix 512 × 512, field of view (FOV) of 400 mm (Brilliance 16 scanner) and 500 mm (other machines), slice thickness of 1-2 mm, the iterative reconstruction algorithm. All CT images were obtained in the supine position during inspiratory breath-hold.

### Imaging Analysis

Two experienced radiologists analyzed the CT images independently with a lung window (window width, 1500 HU; window level, −500 HU) and mediastinum window (window width, 400 HU; window level, 60 HU). Consensus was reached by discussion in case of disagreement. Image features included the following (1): tumor density (solid/part-solid) (2); maximum diameter (3); margin (lobulated, spiculated) (4); air bronchogram (5); pleura indentation, pleural attachment, or pleural closeness (6); distance from the pleura. A part-solid nodule was defined as a tumor that included both GGO and solid components (0<CTR<1.0). A pure-solid nodule was defined as a tumor that included only consolidation without GGO (CTR=1.0) (16). In the current study, pure GGO was excluded since VPI was never observed in these lesions due to its minimally invasive nature and inability to penetrate the thick elastic layer (17). Pleural indentation was defined as tumor indentation of the visceral pleura on CT images at the lung window. Pleural attachment was defined as no visible space between the nodule and the visceral pleura on CT images at the lung window or tumor attachment to the interlobar pleura at the lung window. Pleural closeness was defined as tumor located within 1.0 cm of the pleura (16).

### Histologic Evaluation

Surgically resected specimens were stained with hematoxylin and eosin, and examined to determine the presence or absence of VPI. VPI was defined as invasion beyond the elastic layer of the visceral pleura according to the 8th edition of the TNM classification criteria (5). Histologic evaluation was performed by one experienced pathologist.

### Tumor Segmentation and Radiomics Feature Extraction

**Figure 1:**
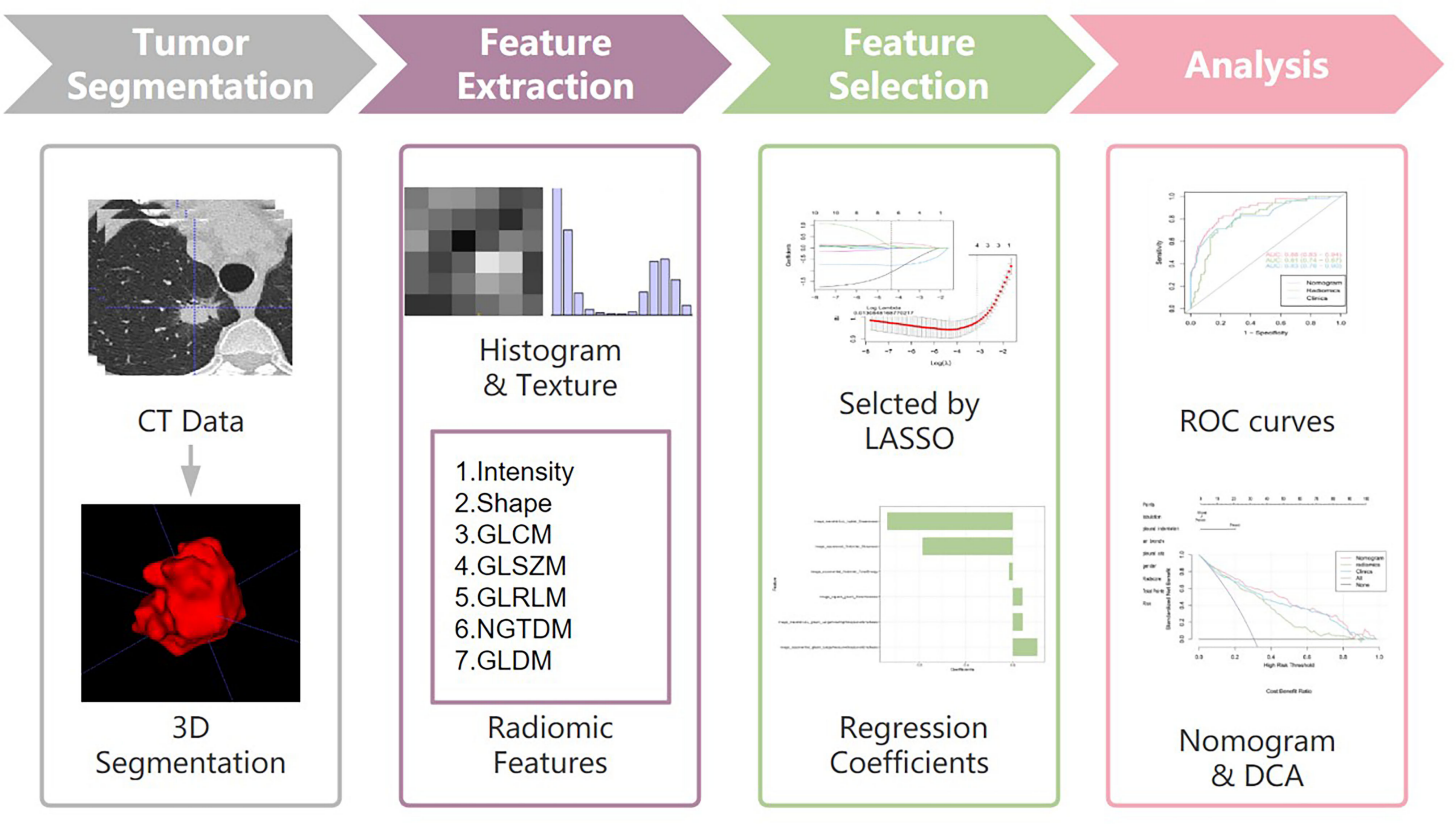
Workflow of the study. Workflow can be divided into four parts: tumor segmentation, feature extraction, feature selection and analysis.

CT images of enrolled patients were exported from the picture archiving and communication system (PACS), and segmented semi-automatically using ITK-SNAP software (version 3.6.0, www.itk-snap.org) (18). The workflow of the analysis is summarized in **Figure 1**. All images were automatically segmented and adjusted by a radiologist with 8 years of experience. After 4 weeks this radiologist segmented the images of 30 randomly selected patients for intra-observer reproducibility. In addition, another radiologist with 20 years of experience segmented 30 randomly selected patient images for inter-observer reproducibility. The inter- and intra-observer reproducibility of feature extraction was evaluated by intraclass correlation coefficients (ICCs). ICCs greater than 0.75 were considered as good consistency.

All images were performed image normalization before feature extraction (19). Radiomics features were extracted from the ROI by the pyradiomic package Python software (version 3.7.12, www.python.org). A total of 1316 high-dimensional features were extracted from each sample and these were classified into seven categories: first order statistics (n = 252), shape (n = 14), neighborhood gray-tone difference matrix (n = 70), grey level dependence matrix (GLDM) (n = 196), grey level co-occurrence matrix (GLCM) (n = 336), run-length matrix (RLM) (n = 224), and grey level zone size matrix (GLZSM) (n = 224).

### Radiomics Feature-Based Prediction Model Construction

Radiomics signature model based on selected features from the training cohort was constructed. Two feature selection methods were used to select the features. First, maximum relevance minimum redundancy (mRMR) was performed to eliminate redundant and irrelevant features. Then, least absolute shrinkage and selection operator (LASSO) was used to select the most useful features. A radiomics score (Radscore) was computed for each patient through a linear combination of selected features weighted by their respective coefficients. The final formula for the Radscore was as follows: “Radscore=0.085*image_wavelet-LLL_glszm_LargeAreaHighGrayLevelEmphasis+-0.034*image_exponential_firstorder_TotalEnergy+-1.071*image_wavelet-LLL_ngtdm_Coarseness+0.21*image_exponential_glszm_LargeAreaLowGrayLevelEmphasis+0.083*image_square_glszm_ZoneVariance+-0.771*image_squareroot_firstorder_Skewness + -1.266”. Furthermore, the Radscore was compared between lung adenocarcinoma with VPI and those without VPI in both the training and validation cohorts.

Logistic regression was performed to select the independent clinical predictors in the training cohort. Prediction models combining radiomics features and clinical variables were established. Finally, a radiomics nomogram based on the multivariate logistic regression model in the training cohort was constructed, and receiver operating characteristic (ROC) curves were developed to evaluate the discriminatory ability of the nomogram. The calibration curve and Hosmer-Lemeshow test was used to assess the goodness-of-fit of nomogram (20, 21). Decision curve analysis was performed to assessed the clinical value of nomogram. The net benefit is calculated within a threshold probability, defined as the minimum probability of a disease requiring further intervention (22).

### Statistical Analysis

Statistical analyses were performed using R software (version 4.1.0) for quantitative characterisation. The characteristics of patients with VPI and without VPI were compared by Student’s t-test for normally distributed data, otherwise the Mann-Whitney u-test was used. The intra-observer reproducibility of tumor segmentation and feature extraction were evaluated by intraclass correlation coefficients (ICCs). ICCs greater than 0.75 were considered to have good consistency. A multivariate binary logistic regression was implemented using the “rms” package. The nomogram was created and the calibration plots were created using the “rms” package. ROC curves were plotted to evaluate the diagnostic efficiency of the nomogram model. The area under the ROC curve (AUC) was calculated. P-values < 0.05 were considered to be significant.

## Results

### Clinical Characteristics

A total of 659 patients were included in this study, of whom 193 (29.3%) were diagnosed with VPI and 466 (70.7%) were diagnosed without VPI (**Table 1**).

**Table 1:** Characteristics of 659 lung adenocarcinoma patients, according to the presence of the visceral pleural invasion.

Characteristics	Total (n=659)	Univariate logistic regression			Multivariate logistic regression
		VPI (−) (n=466)	VPI (+) (n=193)	*P* value	*P* value
**Gender**				**<0.001**	**0.01**
Male	244	150	94		
Famale	415	316	99		
**Age(years)**	61 (53-67)	60 (52-66)	63 (57-69)	**<0.001**	NA
**Smoking status**				**<0.001**	NA
Active	553	413	140		
Inactive	106	53	53		
**lobulation**				**<0.001**	**0.04**
Present	481	315	166		
Absent	178	151	27		
**spiculation**				0.528	
Present	479	342	137		
Absent	180	124	56		
**air bronchogram**				**<0.001**	**<0.001**
Present	352	298	54		
Absent	307	168	139		
**Radiological tumor type**				**<0.001**	NA
Pure-solid	381	217	164		
Part-solid	278	249	29		
**Pleura indentation**				**<0.001**	**<0.001**
Present	422	266	156		
Absent	237	200	37		
**Pleural attachment**				**<0.001**	**<0.001**
Present	327	193	134		
Absent	332	273	59		

Age is expressed as Median (interquartile range). Otherwise, data are number of patients. The P value marked bold indicated statistical significance.

NA means that the characteristic is not included in the logistic regression.

There were significantly differences between VPI-presence and VPI-absence group in gender, pleural indentation, pleural attachment, air bronchogram, and lobulation (all P < 0.05). Gender, pleural indentation, pleural attachment, air bronchogram, and lobulation were independent risk factors for predicting VPI after logistic regression analysis (**Table 2**). A clinical model was developed based on these characteristics.

**Table 2:** Variables and coefficients of clinical model.

Variable	Adjusted OR	95%CI	*P* value
Gender	1.95	1.17-3.25	0.011
Lobulation	0.32	0.19-0.53	0.040
Air bronchogram	2.11	1.03-4.31	<0.0001
Pleura indentation	19.07	9.38-38.76	<0.0001
Pleural attachment	10.10	5.33-19.14	<0.0001

OR, odds ratio; CI, confidence interval.

### Reproducibility Analysis

The average ICCs of intra-observer was 0.96, indicating satisfactory agreement. The number of features with fair consistency (0.75 > ICC ≥ 0.4) and poor consistency (ICC <0.4) were 4 (0.3%) and 26 (2.0%), respectively. The average ICCs of inter-observer was 0.95, indicating satisfactory agreement. The number of features with fair consistency (0.75 > ICC ≥ 0.4) and poor consistency (ICC <0.4) were 11 (0.8%) and 32 (2.4%), respectively.

### Radiomics Feature Selection Signature Construction, Validation, and Evaluation

**Figure 2:**
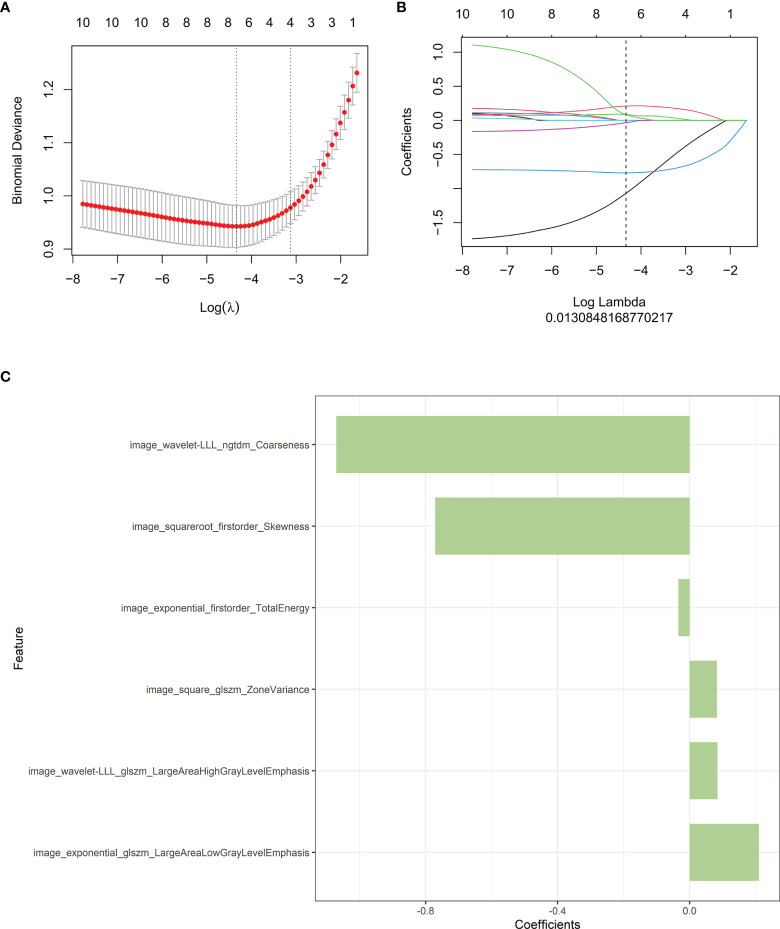
Radiomics features associated with VPI were selected using LASSO regression models. **(A)** Cross-validation curve. An optimal log lambda (0.013) was selected, and 6 non-zero coefficients were chosen. **(B)** LASSO coefficient profiles of the 1316 radiomics features against the deviance explained. **(C)** Histogram shows the contribution of the selected parameters with their regression coefficients in the signature construction.

**Figure 3:**
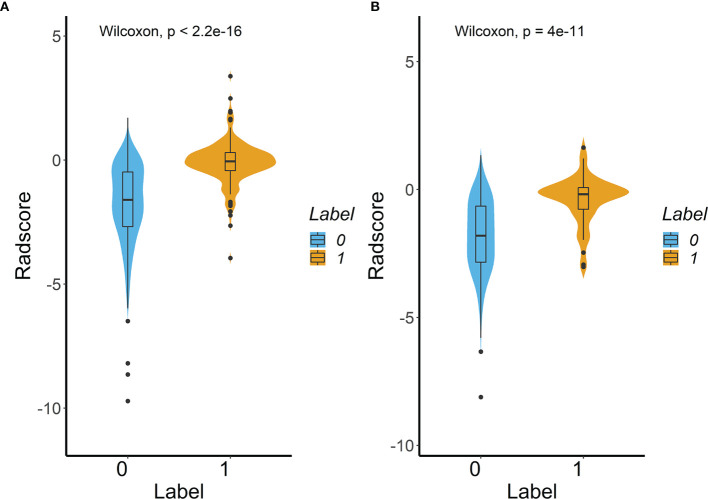
Difference in the Radscore between lung adenocarcinoma with VPI and without VPI in training cohort **(A)** and validation cohort **(B)**. (Label 0: No VPI; label 1: VPI).

30 features were retained after eliminating the redundant and irrelevant features with mRMR. Then, 6 features were selected as the most predictive subset after LASSO (**Figures 2A, B**). The corresponding coefficients were evaluated (**Figure 2** **C**) and a predictive model was constructed. Radscore was calculated by summing the selected features weighted by their coefficients. There was a significant difference in radscore between lung adenocarcinoma with VPI and without VPI in the training and validation groups (**Figure 3**).

**Figure 4:**
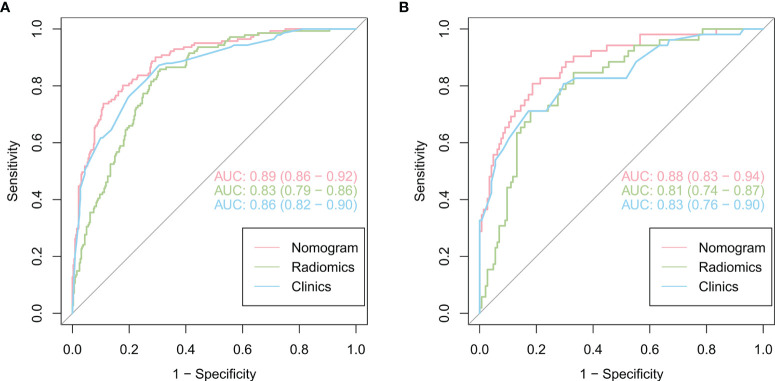
Comparison of the performance of three models for predicting VPI in lung adenocarcinoma. ROC curves for clinical features alone, radiomics features alone and combined features for the training **(A)** and validation **(B)** cohorts.

As shown in **Figure 4**, the radiomics feature model had an AUC of 0.83 in the training cohort and 0.81 in the validation cohort. Then, clinical indicators (**Table 1**) with p values less than 0.01 in the logistic regression analysis with radscore were used to constructed a combined model, which showed an AUC of 0.89 (95% CI, 0.86-0.92) in the training cohort (**Figure 4A**) and an AUC of 0.88 (95% CI, 0.83-0.94) in the validation cohort (**Figure 4B**). The predictive performance of the combined model was shown in **Table 3**. In both the training and validation cohorts, the accuracy, specificity, positive predictive value, and AUC of the combined model outperformed both the radiomics feature model and the clinical feature-based model.

**Table 3:** Predictive performance of the three models in the training and validation cohorts.

Model	Accuracy [95%CI]	AUC [95%CI]	Sensitivity	Specificity	PPV	NPV
**Training cohort**						
Radiomics features	0.74 [0.70-0.78]	0.83 [0.79-0.86]	0.86	0.69	0.55	0.91
Clinics features	0.75 [0.71-0.79]	0.86 [0.82-0.90]	0.87	0.69	0.56	0.93
Joint features	0.84 [0.81-0.88]	0.89 [0.86-0.92]	0.74	0.89	0.75	0.89
**Validation cohort**						
Radiomics features	0.73 [0.66-0.79]	0.81 [0.74-0.87]	0.75	0.72	0.49	0.88
Clinics features	0.73 [0.66-0.79]	0.83 [0.76-0.90]	0.79	0.71	0.49	0.90
Joint features	0.83 [0.78-0.89]	0.88 [0.83-0.94]	0.71	0.88	0.65	0.90

AUC, area under the curve; 95%CI, confidence interval; PPV, positive predictive value; NPV, negative predictive value.

**Figure 5:**
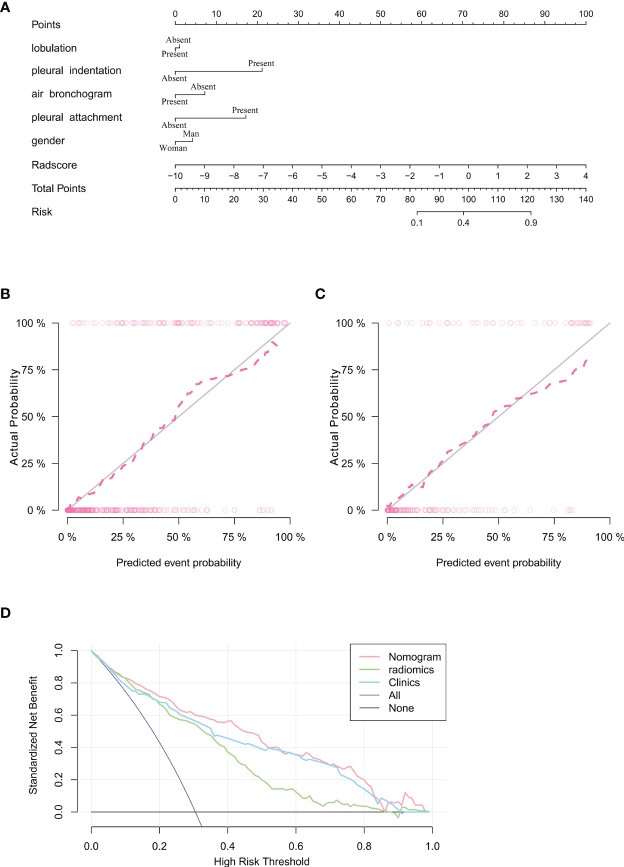
Nomogram for prediction of VPI based on training cohort and the model evaluation of calibration curve. **(A)** Radiomics nomogram based on clinical characteristics and Radscore. The calibration curves were used to evaluate the consistency of the probability of VPI predicted by the nomogram with the actual fraction of visceral pleural invasion in the training **(B)** and validation **(C)** cohorts. **(D)** DCA for the prediction of VPI in lung adenocarcinoma for each model. X-axis represents the threshold probability and Y-axis represents the net benefit. The red curve represents the nomogram. The blue curve represents the clinical features model. The green curve represents the radiomics features model.

Subsequently, a nomogram model was created (**Figure 5A**). The calibration curve of the nomogram for predicting VPI matched well with the estimated and actual observed values of the radiomics nomogram. The p-value for the predictive power of the nomogram obtained by the Hosmer-Lemeshow test was 0.94 in the training cohort (**Figure 5B**) and 0.86 in the validation cohort (**Figure 5C**).

The DCA showed that the net benefit of the combined nomogram outperformed the clinical and radiomics feature models (**Figure 5D**). The decision curve showed that the combined nomogram established in this study has more benefit for predicting VPI if the threshold probability of a patient is between 0 to 55%, 60% to 80% and 90% to 100%.

## Discussion

This study constructed and validated a nomogram model based on clinical and radiomics features extracted from CT imaging for identifying VPI in lung adenocarcinoma less than or equal to 3 cm. The nomogram model was able to classify stage I lung adenocarcinoma into those with VPI and without VPI, with AUC values greater than those of the radiomics model and the clinical model. The results demonstrated that the combined model can reliability predict VPI.

In this study, we included patients with lung adenocarcinoma to construct a nomogram to predict with or without VPI. This is because research showed that no significant difference in survival rates associated with VPI in NSCLC (23). As for NSCLC, squamous cell carcinoma and adenocarcinoma showed significantly different biological behaviors (24). Whereas the heterogeneity in biological behavior between lung adenocarcinoma and squamous cell carcinoma can be reflected by radiomics, radiomics can predict their histological subtypes (25, 26). In addition, lung adenocarcinoma is the most common subtype of lung cancer, so in this study we only discussed lung adenocarcinoma.VPI is a poor prognostic factor for lung adenocarcinoma (27–29), since VPI has been associated with increased overall mortality and decreased disease-free survival (30). The visceral pleura is rich in lymphatic vessels and forms an intercommunicating network on the lung surface. This network penetrates the lung parenchyma, connects to the bronchial lymphatics and flows into the hilar lymph nodes (31), which may progressively develop into metastatic disease (lymph node metastasis or distant metastasis). According to the 8th edition of the AJCC staging manual, a tumour size of 0-3 cm with VPI (including PL1 and PL2) is considered IB stage (31). Some previous studies have shown that patients with stage IB NSCLC can benefit from adjuvant chemotherapy treatment (32–34).

The correlation between CT morphological features and VPI has been reported previously (30, 35, 36). The present study concluded that lobulation and air bronchogram were not significant indicators of VPI in lung adenocarcinoma, which was consistent with previous study (30, 35). A lobulated contour implies uneven growth, which is associated with malignancy. However, lobulation also occurs in up to 25% of benign nodules (37). In our study, the lobulation sign was not an independent risk factor for predicting VPI, which may be due to a selection bias resulting from the small size of our enrolled tumors and the fact that the number of patients in this study was not large enough. The air bronchogram sign is the result of tumor cells spreading along the wall of fine bronchus and alveolar wall in a volvulus-like growth pattern without destroying the lung scaffold structure, and the residual gas in the bronchus and alveoli is visualized (38). In previous studies, air bronchogram signs were associated with low invasiveness and helped to distinguish with or without VPI of lung adenocarcinoma (39). Many studies have suggested that the node-pleura relationship is an important predictor of positive VPI. In lung adenocarcinoma, pleural indentation is generally considered to be a positive predictor of VPI (39). Indentation increases the risk of tumor invasion of the visceral pleura (40). Pleural attachment is another known factor for local recurrence and poor survival of lung adenocarcinoma after radiotherapy for non-small cell lung cancer (41, 42). Although most studies have evaluated the morphologic features of VPI on CT images, the accuracy of studies based on morphologic features of CT images remained low, and the morphological features identified are dependent on the experience of the radiologists (7–10).

Currently, radiomics allows for the non-invasive evaluation of internal tumor heterogeneity by extracting and analyzing a large number of advanced quantitative imaging features from CT images (12, 43). Yuan et al. proposed a support vector machine (SVM) based deep learning model to predict the status of VPI from preoperative CT scans, with a high AUC in the validation cohort (10). However, the model could only distinguish patients with or without VPI based on radiomics models, without incorporating relevant clinical characteristic parameters, and did not take the relations of tumor to adjacent pleura into account.

In this study, six optimal quantitative radiomics features (including Coarseness, Skewness, TotalEnergy, ZoneVariance, LAHGLE and LALGLE) were extracted Coarseness is a parameter for the neighbouring gray tone difference matrix (NGTDM). The lower coarseness values in the present study indicated more heterogeneous textures of the lesion. Skewness and total energy are both the first order parameters. Lower skewness and total energy values indicated higher heterogeneity of the lesion. LAHGLE and LALGLE are parameters for the gray level size zone matrix (GLSZM). Zone variance is also a parameter for the GLSZM. In this study, the high LAHGLE, LALGLE and zone variance values indicated high heterogeneity of the lesion.

According to our findings, the model that combines radiomics features and clinical features is more effective.The clinical features model included four semantic features (the signs of lobulation, air bronchogram, pleural attachment and pleural indentation) among the five features, all describing the perimeter and morphology of tumor. Because of the difficulty in outlining peritumoral ROI due to the proximity of the tumor to the pleura, this imaging histology study focused only on the interior of the tumor. However, we included some of the imaging signs to reflect the peritumoral situation as described previously. Previous studies have demonstrated that the above features were the risk factors of VPI. Furthermore, the radiomics focused on the heterogeneity within the tumor, the two models were complementary to each other. Although the diagnostic performances of the radiomic model and the clinical model were similar, the combination of the two models can obtain a higher diagnostic efficiency. The AUC values of the combined model were higher than those of the radiomics and clinical models (p < 0. 05). Moreover, the DCA results showed that the nomogram was superior to both the clinical features model and the radiomics model for most ranges of reasonable threshold probabilities.

There are several limitations in this study. Firstly, it was a retrospective study and there may have been selection bias. Secondly, tumour serum indicators may be missing due to the small size of the tumour. Thirdly, multiple different CT scanning devices were used, using different acquisition protocols. Multicentre studies should be conduted to validate the reliability of Nomogram.

In summary, a CT image-based nomogram model combining radiomics features and clinical features was developed for predicting VPI in lung adenocarcinoma. A nomogram based on radiomics features may provide a non-invasive method to evaluate the prognosis of early lung adenocarcinoma.

## Data Availability Statement

The raw data supporting the conclusions of this article will be made available by the authors, without undue reservation.

## Author Contributions

XZ and CH designed the research. XZ and JB helped collect patient information. YL and XP analyzed data. YL and QW prepared figures and tables. XZ and YL wrote the paper. CH and SH conceived the project and supervised and coordinated all aspects of the work. All authors contributed to the article and approved the submitted version.

## Funding

This study was supported by Gusu health talent project of Suzhou (GSWS2020003).

## Conflict of Interest

The authors declare that the research was conducted in the absence of any commercial or financial relationships that could be construed as a potential conflict of interest.

## Publisher’s Note

All claims expressed in this article are solely those of the authors and do not necessarily represent those of their affiliated organizations, or those of the publisher, the editors and the reviewers. Any product that may be evaluated in this article, or claim that may be made by its manufacturer, is not guaranteed or endorsed by the publisher.
